# Are mast cells instrumental for fibrotic diseases?

**DOI:** 10.3389/fphar.2013.00174

**Published:** 2014-01-21

**Authors:** Catherine Overed-Sayer, Laura Rapley, Tomas Mustelin, Deborah L. Clarke

**Affiliations:** Department of Respiratory, Inflammation and Autoimmunity, MedImmune LtdCambridge, UK

**Keywords:** fibrosis, mast cells, lung, idiopathic pulmonary fibrosis, TGF-β

## Abstract

Idiopathic pulmonary fibrosis (IPF) is a fatal lung disorder of unknown etiology characterized by accumulation of lung fibroblasts and extracellular matrix deposition, ultimately leading to compromised tissue architecture and lung function capacity. IPF has a heterogeneous clinical course; however the median survival after diagnosis is only 3–5 years. The pharmaceutical and biotechnology industry has made many attempts to find effective treatments for IPF, but the disease has so far defied all attempts at therapeutic intervention. Clinical trial failures may arise for many reasons, including disease heterogeneity, lack of readily measurable clinical end points other than overall survival, and, perhaps most of all, a lack of understanding of the underlying molecular mechanisms of the progression of IPF. The precise link between inflammation and fibrosis remains unclear, but it appears that immune cells can promote fibrosis by releasing fibrogenic factors. So far, however, therapeutic approaches targeting macrophages, neutrophils, or lymphocytes have failed to alter disease pathogenesis. A new cell to garner research interest in fibrosis is the mast cell. Increased numbers of mast cells have long been known to be present in pulmonary fibrosis and clinically correlations between mast cells and fibrosis have been reported. More recent data suggests that mast cells may contribute to the fibrotic process by stimulating fibroblasts resident in the lung, thus driving the pathogenesis of the disease. In this review, we will discuss the mast cell and its physiological role in tissue repair and remodeling, as well as its pathological role in fibrotic diseases such as IPF, where the process of tissue repair and remodeling is thought to be dysregulated.

## INTRODUCTION

**FIGURE 1:**
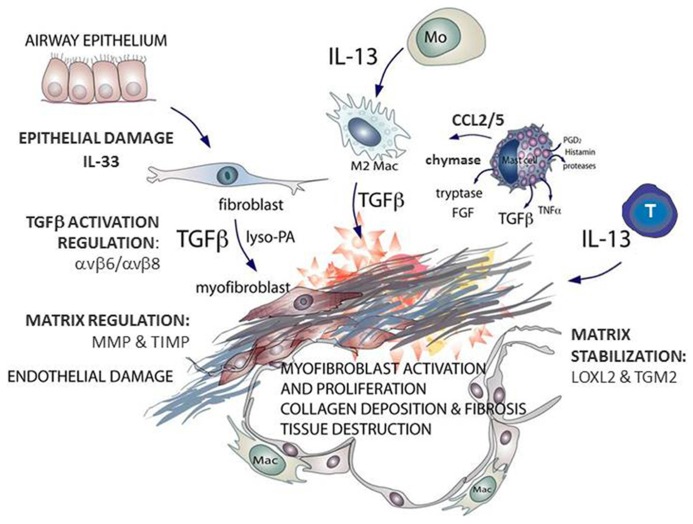
**Schematic outlining the key mast cell mediators contributing to fibrosis, the effector cells that generate transforming growth factor (TGF)β and interleukin (IL)-13 and the enzymes associated with idiopathic pulmonary fibrosis (IPF).** Multiple cell types are found at sites of lung fibrosis. Many are direct producers of extracellular matrix (ECM), or indirectly promote the generation and deposition of aberrant matrix. Mast cells generate pro-fibrotic mediators including tryptase, chymase, and chemokines that promote fibroblast activation. This figure is adapted from Clarke et al. (2013).

Idiopathic pulmonary fibrosis (IPF) is a lethal lung disorder of unknown etiology. With a median survival of 3–5 years and an increasing incidence (Dempsey, 2006; Navaratnam et al., 2013) IPF is attracting much research attention. Histologically, IPF is characterized by heterogeneity: areas of normal parenchyma are interspersed with areas of paraseptal and subpleural fibrosis (Raghu et al., 2011). The fibrotic process is often defined as an aberrant wound healing response, involving dysregulated tissue repair and remodeling (Wynn, 2011). There is epithelial damage, inflammatory cell infiltration, elevated pro-fibrotic cytokine expression, formation of fibroblastic foci, and increased extracellular matrix (ECM) deposition (Wilson and Wynn, 2009; **Figure 1**).

The precise link between inflammation and fibrosis remains unclear, but it appears that immune cells can promote fibrosis by releasing fibrogenic factors (Stramer et al., 2007). So far, however, therapeutic approaches targeting macrophages, neutrophils, or lymphocytes have failed to alter disease pathogenesis. A new cell to garner research interest in fibrosis is the mast cell. Indeed, increased numbers of mast cells have long been known to be present in pulmonary fibrosis (Kawanami et al., 1979), and clinically correlations between mast cells and fibrosis have been reported (Metcalfe et al., 1997). More recent data suggests that mast cells may drives the fibrotic process by stimulating fibroblasts resident in the lung (Wygrecka et al., 2013), thus driving the pathogenesis of the disease. In this review, we will discuss the mast cell and its physiological role in tissue repair and remodeling, as well as its pathological role in fibrotic diseases such as IPF, where tissue repair and remodeling are dysregulated (Wynn, 2011). Additionally, while the inflammatory roles of mast cells in asthma and atopic dermatitis are well characterized (Dougherty et al., 2010; Andersson et al., 2011a), the potential role of mast cells in mediating the tissue remodeling components of these diseases is also discussed.

## MAST CELL BIOLOGY

Mast cells are innate immune cells, originally named by Paul Ehrlich in 1878 on the basis of their granular histological staining with aniline dyes. Both human and rodent mast cells originate from hematopoietic stem cells (HSCs) in the bone marrow, which give rise to common myeloid progenitors that can subsequently differentiate into mast cell precursors (Rodewald et al., 1996; Austen and Boyce, 2001; Franco et al., 2010). A number of publications suggest that mast cells could also arise from a shared basophil/mast cell progenitor. These cells had until recently only been identified in the mouse spleen (Arinobu et al., 2005), but have now also been identified in mouse bone marrow (Qi et al., 2013). In humans, common progenitor cells have been identified in the context of disease, for example in patients with myeloproliferative diseases (Zucker-Franklin, 1980). The exact origin of mast cells remains controversial, and others suggest that mast cells diverge from basophils relatively early in their differentiation (Chen et al., 2005; Mukai et al., 2012). Mast cells circulate as somewhat immature cells (Kitamura et al., 1979) and undergo final maturation upon entry into peripheral tissues. They reside within most tissues of the body, and are notable at sites that act as barriers to the external environment such as the skin, lung, and the intestine.

The phenotypic heterogeneity of mast cells is well known, with two major subsets being recognized. In rodents, the two subsets have different anatomical localization and expression pattern of proteases. Connective tissue mast cells (CTMCs), as the name implies, are mainly located in the connective tissues of the intestinal submucosa, the peritoneal cavity and surrounding blood vessels, and in the skin. These cells express both chymases and tryptases. In contrast, mucosal mast cells (MMCs) are usually found in the mucosal tissues of the lung and intestine and mainly express chymases (Gurish and Austen, 2012). In humans, the two subtypes of mast cells can similarly be characterized on the basis of their protease expression: MC_T_ express tryptase only whereas MC_TC_ contain both tryptase and chymase. The former appear to correspond to the murine mucosal type mast cell and the latter to CTMCs (Irani et al., 1986; Welle, 1997).

In addition to differences in protease content and anatomical localization, mast cells are also heterogeneous with regard to receptor expression and mediator release, and in their functional responses to various stimuli (Lowman et al., 1988; Bradding et al., 1995; Andersson et al., 2009). Some of the more well-characterized receptors are those required for the survival and/or activation of mast cells. Activation of the receptor c-kit is required for the survival, proliferation and differentiation of all mast cells via its ligand stem cell factor (SCF). A well characterized mechanism of mast cell activation is through aggregation of FcεR1, the high affinity IgE receptor (Razin et al., 1983), although stimulation of other receptors, such as TLRs and the IL-33 receptor ST2 can also result in mast cell activation (Smith and Parikh, 1999; Varadaradjalou et al., 2003).

Upon activation, mast cells secrete an array of biologically active mediators (reviewed in Gilfillan and Beaven, 2011). Rapid mediator release occurs within minutes from pre-formed granules which can be completely emptied through exocytosis or partly emptied, but preserved for renewed release. Granules contain histamine, lipid mediators, proteoglycans, and proteases like chymases, tryptases, and carboxypeptidases (Riley and West, 1953; Hogan and Schwartz, 1997; Stevens and Adachi, 2007). Activated mast cells can also generate eicosanoids (leukotrienes and prostaglandins) by enzymatic biosynthesis from arachidonic acid (Boyce, 2005). They can also synthesize and release a wide array of cytokines, chemokines and growth factors, including but not limited to IL-3, IL-4, IL-5, IL-6, IL-10, IL-13, GM-CSF, TNF-α, TGF-β, CCL2, CCL5, fibroblast growth factor (FGF) and platelet derived growth factors (PDGF; Burd et al., 1989, 1995; Plaut et al., 1989; Lukacs et al., 1996; Kanbe et al., 1999; Oliveira and Lukacs, 2001; Artuc et al., 2002), which occurs over a longer time-frame of hours rather than minutes, with the exception of TNF-α which may be stored and released from granules (Gordon and Galli, 1990).

The wide array of mediators that can be released from mast cells may be an indication of the versatility of mast cells and indicate that they may have many distinct effector functions. However, studying mast cell function is challenging due to the rare nature of the cells in circulation (particularly in healthy individuals) and the difficulty of isolating mast cells without affecting their activation state. In humans there are examples of rare disorders resulting from enhanced mast cell mediator release, namely systemic mast cell activation disease (MCAD). There are three forms of MCAD, the most prevalent is mast cell activation syndrome (MCAS), followed by systemic mastocytosis, then mast cell leukemia (Haenisch et al., 2012). Additionally, MCAS has been implicated as an underlying cause of fibromyalgia (Lucas et al., 2006) and irritable bowel syndrome (Frieling et al., 2011). There are however, no reports of humans with mast cells deficiencies to aid in the deciphering of their physiological roles in human physiology. In contrast, mast cell-deficient mouse strains have been used extensively to study mast cell function. However, these strains generally have loss of function mutations in the c-kit – SCF axis, complicating the picture with deficiencies in other c-kit expressing hematopoietic cells. More recently, new mouse models have been developed and will likely give a clearer picture of the functions unique to mast cells (Reber et al., 2012).

Traditionally, mast cells are thought to have evolved to confer protection against pathogens, particularly parasitic and fungal infections (Abraham and St John, 2010). Carboxypeptidase3, a mast cell product, can degrade the snake venom toxin safarotoxin (Metz et al., 2006). Other characterized effects include protection against infections with gastrointestinal helminths as well as acquired tick resistance (reviewed in Voehringer, 2013). In addition to protection against infection, other physiological roles for mast cells involving the regulation of the innate and adaptive immune system have been reported. These include tolerance against skin graft rejection, angiogenesis, as well as tissue remodeling and wound healing (Gilfillan and Beaven, 2011). The role in tissue repair and remodeling is described here, given the relevance to the pathological role of mast cells in fibrosis which is the main subject of this review.

Upon injury, there are several stages of the wound healing process; inflammation, fibroblast migration/proliferation, and remodeling. Although the pathogenesis of IPF is unclear, it is thought to involve recurrent injury to the airway epithelium and/or an aberrant wound healing response, and the stages of fibroblast proliferation/migration and tissue remodeling are particularly relevant to the fibrotic process (Wynn, 2011). Although studies of mast cell-deficient mice have reported conflicting data on the effect of mast cell deficiency on wound closure, most studies reported that mast cell depletion delayed, but did not prevent, tissue repair and remodeling (Egozi et al., 2003; Iba et al., 2004; Weller et al., 2006; Gallant-Behm et al., 2008; Shiota et al., 2010; Younan et al., 2011; Antsiferova et al., 2013). There is evidence that mast cells participate in multiple stages of the wound healing process (reviewed in Wulff and Wilgus, 2013). Within days of injury, mast cells accumulate along the edges of the wound (Trautmann et al., 2000). They likely contribute to local inflammation by stimulation of vascular permeability through secretion of histamine, lipid mediators, and VEGF, as well as through recruitment of other cell types (e.g., neutrophils via chymase; Dvorak, 2005; Weller et al., 2006; Takato et al., 2011). Although there are other aspects of wound healing (e.g., apoptosis) the processes of repair and remodeling are particularly relevant to fibrosis. Mast cells have been shown to stimulate the migration and proliferation of fibroblasts (Levi-Schaffer and Kupietzky, 1990), a key cell type in the proliferative phase of the wound healing process. These effects are mediated at least in part by cytokines and growth factors released by mast cells, including keratinocyte growth factor epidermal growth factor and platelet-derived growth factor (PDGF; Artuc et al., 1999, 2002; Noli and Miolo, 2001; Voehringer, 2013), as well as histamine and tryptase (described in more detail below; Levi-Schaffer and Piliponsky, 2003).

## MAST CELL PRODUCTS AS MEDIATORS OF REMODELING

Many of the mast cell products described above as mediators of normal physiological processes, if released in excess, can trigger allergic and anaphylactic responses, and therefore also contribute to a well characterized pathological role for mast cells. In contrast to this well-established role of mast cells in acute allergy and anaphylaxis, their role in diseases involving chronic remodeling processes is much less clear. Several mast cell products are pro-fibrotic including TGF-β, IL-13, CCL2, CCL5, IL-4, PDGF, and FGF (and this list is not exhaustive). The roles of some of these mediators in fibrotic processes are described in more detail below.

### TGF-β

Although mast cells are not the sole source of TGF-β1, this pleiotropic cytokine is perhaps the most well characterized pro-fibrotic mediator produced by mast cells. TGF-β has multiple functions that contribute to its role in fibrosis, including actions upon fibroblasts and myofibroblasts, key cell types involved in the pathogenesis of fibrosis. For example, TGF-β promotes fibroblast migration and proliferation (Postlethwaite et al., 1987), as well as collagen formation and fibroblast differentiation into myofibroblasts (Fine and Goldstein, 1987; Goldstein et al., 1992; Desmouliere et al., 1993). Studies also suggest a role in epithelial–mesenchymal transition (EMT; Willis et al., 2005).

### TRYPTASE/CHYMASE

Mast cell proteases tryptase and chymase have effects on both the connective tissue cells involved in fibrosis as well as the surrounding ECM. These proteases stimulate proliferation of airway smooth muscle cells, epithelium and fibroblasts, as well as fibroblast chemotaxis and myofibroblast differentiation (Ruoss et al., 1991; Brown et al., 1995; Cairns and Walls, 1996; Gruber et al., 1997). Mast cell tryptase can also stimulate the synthesis of type I collagen by fibroblasts (Cairns and Walls, 1997; Gruber et al., 1997), however, both proteases can also activate matrix metalloproteinases, and may therefore increase turnover of the ECM (Tchougounova et al., 2005; Iddamalgoda et al., 2008). Tryptase-mediated stimulation of fibroblast proliferation occurs via activation of the protease activated receptor PAR-2 (Akers et al., 2000), and more recently a PAR-2 dependency has also been demonstrated for tryptase-induction of collagen and fibronectin synthesis by fibroblasts (Wygrecka et al., 2013). Furthermore, these authors hypothesized that the increase in PAR-2 expression observed in lung fibroblasts from patients with IPF could sensitize these cells to the effects of mast cell-derived tryptase (Wygrecka et al., 2013).

### IL-13

The Th2 cytokine IL-13 is a pro-fibrotic mediator that is thought to promote fibrosis via TGF-β -dependent and independent mechanisms. IL-13 can induce TGF-β production and activation *in vivo* and in human airway fibroblasts and this is thought to involve remodeling through IL-13Rα2 (Lee et al., 2001; Fichtner-Feigl et al., 2006; Firszt et al., 2013). IL-13 can also directly promote fibrosis by stimulating proliferation or collagen production by fibroblasts as well as differentiation into myofibroblasts (Oriente et al., 2000; Saito et al., 2003; Ingram et al., 2004).

### CC CHEMOKINES

CCL2 is a chemokine that signals through the receptor CCR2. In addition to displaying chemotactic activity for immune cells such as monocytes, a role in fibrosis is suggested by the ability to attract fibrocytes to the airways following lung injury (Kay, 2005). Furthermore, CCL2 can stimulate fibroblast collagen production via up-regulation of TGF-β expression (Holgate, 2008). The interplay between TGF-β , IL-13, and CCL2 in the context of fibrosis is discussed in more detail in (Manuyakorn et al., 2013). As well as being synthesized by mast cells (Lukacs et al., 1996), CCL5 also acts as a mast cell chemoattractant (Mattoli et al., 1995). While the role of CCL5 as a fibrotic mediator is less clear compared to that of CCL2, there is some evidence that antagonism of CCL5 may be therapeutic in liver fibrosis, possibly through the modulation of monocyte subpopulations (Berres et al., 2010; Stock et al., 2013).

## MAST CELLS IN DISEASE

Mast cells are key contributors to multiple diseases in which there is an element of tissue remodeling, of which asthma and atopic dermatitis are two.

### ASTHMA

Asthma is traditionally an inflammatory airway disease where patients present with airflow obstruction caused by airway narrowing, an increase cellular infiltrate (eosinophils, neutrophils, T cells) to the lung and mucus plugging of the airways. The inflammation is typically Th2 driven and eosinophilic (Kay, 2005) involving many of the mediators mentioned previously. These are useful disease indictors to guide treatment; however this mechanism does not explain all aspects of asthma. There are fundamental structural changes in the asthmatic lung. The inability of anti-inflammatory treatments to reverse symptoms or the decline in lung function (Holgate, 2008) in some asthmatics is suggestive of a mechanism of uncontrolled airway remodeling significantly contributing to disease pathology (Manuyakorn et al., 2013).

Many structural changes occur in asthma, including epithelial shedding, enlarged submucosal glands, subepithelial basement membrane thickening and fibrosis as well as increased smooth muscle (Manuyakorn et al., 2013). The most striking change is in the smooth muscle which increases in amount by hyperplasia and hypertrophy, as well as spreading up and down the airway (James and Carroll, 2000), a mechanism for which remains unknown (James et al., 2005). Increasing smooth muscle contributes to airway wall thickness which is also driven by deposition of extra cellular matrix including collagen (Black et al., 2003; Howarth et al., 2004).

Mast cells have been shown to be increased in asthma (Dougherty et al., 2010; Andersson et al., 2011b). In the lung the predominant mast cell is MC_T_ (Irani et al., 1986), however MC_TC_, normally present in low numbers, increase with asthma severity (Balzar et al., 2011). The normal and asthmatic airways contain similar numbers of mast cells in the submucosal connective tissues, however there are increased mast cells in the epithelial layer and smooth muscle (Brightling et al., 2002; Kaur et al., 2006), as well as the bronchoalveolar lavage (BAL) fluid of patients with asthma (Shindoh et al., 1987). Increased mast cell mediators in the BAL fluid (Gibson et al., 1993) support the theory of increased presence but also increased reactivity of asthmatic mast cells over non-asthmatic mast cells. The IL-33/ST2 axis has proved to be key in mast cell biology. Mast cells are one of the highest ST2 expressing hemopoietic cell types (Moritz et al., 1998), ST2 is the receptor for the IL-1 family member, IL-33 (Schmitz et al., 2005). IL-33 is an alarmin released upon cell injury (Enoksson et al., 2011) and could therefore play a critical role in wound healing and remodeling. Both IL-33 and ST2 have been shown to be upregulated in asthma (Prefontaine et al., 2009).

The relationship between smooth muscle hyperplasia, fibrosis, and mast cells is unknown although it can be speculated that SCF producing smooth muscle cells sustain mast cell survival (Page et al., 2001), and in turn mast cells mediators such as tryptase (Brown et al., 1995) and histamine (Hollins et al., 2008) could drive this phenomenon.

### ATOPIC DERMATITIS

Atopic dermatitis (AD) is a chronic allergic inflammatory disease affecting both children and adults in which mast cells are thought to be a key cell driving the immune response. The main symptom, pruritis (chronic itching) causes breakdown of the dermal barrier allowing the opportunity for bacterial entry and colonization of skin lesions, persistent inflammation, remodeling and fibrosis.

Many mediators, including histamine and Th2 cytokines that have been reported to be pro-fibrogenic and released from mast cells, are involved in AD (Greaves and Khalifa, 2004; Darsow et al., 2012; Metz et al., 2013). Additionally evidence is emerging for a role for IL-33/ST2 axis in AD (Cevikbas and Steinhoff, 2012).

There appears to be two phases of the disease which progress with severity as well as remodeling with matrix deposition causing toughening/fibrosis of the skin (Hamid et al., 1994). Mast cells are reportedly more activated and undergoing degranulation in mild disease, and increased numbers are associated with severe disease (Hamid et al., 1994). There is evidence of a mast cell switch from a MC_TC_ phenotype (predominant subtype in the skin) to MC_T_ mast cells (Irani et al., 1986).

## ROLE FOR MAST CELLS IN RENAL AND LUNG FIBROSIS

### RENAL FIBROSIS

An association between mast cell recruitment/infiltration and fibrotic diseases has been reported in a number of organs to date. Mast cells are elevated in various kidney disorders, in which the fibrosis is characterized by increased proliferation of fibroblasts and excessive accumulation of ECM (Silver, 2013). Mast cells have been reported to play an integral part of the overall inflammatory process and play a crucial role in interstitial fibrosis in renal amyloidosis (Toth et al., 2000). Liu et al. (2010) recently confirmed these findings, and suggested a role for SCF and PAR-2 in mast cell recruitment and pathology. One study looking at a range of renal diseases revealed that the degree of renal interstitial fibrosis was well correlated with the number of infiltrating tryptase-positive mast cells (Kondo et al., 2001).

In experimental models, mast cells have been reported to be crucial to renal fibrosis induced by ureteral obstruction (Holdsworth and Summers, 2008), supporting the findings from Veerappan et al. (2012) reporting that mast cells are required for the development of renal fibrosis in the rodent unilateral ureteral obstruction model. These reports show causality for mast cells in experimental renal fibrosis and support clinical findings correlating mast cell numbers and the degree of fibrosis obstruction (Holdsworth and Summers, 2008). Additionally, the mast cell remodeling sodium chromoglycate has been reported to attenuate renal fibrosis (Summers et al., 2012), therefore it is proposed that pro-fibrotic mediators released via mast cell degranulation drive fibroblast activation and proliferation, and therefore targeting this cell type in renal disease could be attractive.

The concept of mast cells in pulmonary fibrosis is not a new one. Indeed, in the late ‘80s, and early ‘90s, a number of laboratories identified an increase in mast cells, or their products, in the lungs of IPF patients. In 1986, Rankin and colleagues compared the levels of histamine, a known mast cell product, in the BAL fluid of normal patients versus IPF, asthma, and sarcoidosis and reported increased histamine in IPF samples only (Rankin et al., 1987). In 1987, Shindoh and colleagues reported that when comparing basophilic cells (BCs) in the BAL fluid from patients with asthma and IPF, formalin-insensitive BCs, which were presumed to be CTMCs, were observed in BAL fluids from IPF patients whereas basophils were the major components of BCs in asthmatic patients. These compared to control subjects in which almost all of BCs were MMCs (Shindoh et al., 1987). In 1990, Fortoul and colleagues compared the number of mast cells in the lungs of IPF vs non-fibrotic lung disease vs normal, finding an approximate 10-fold increase in interstitial mast cells compared with the non-fibrotic patients, whereas the mast cell levels were almost equal in both groups of patients in the subpleural, peribronchiolar, and perivascular areas (Fortoul and Barrios, 1990). In 1990, Hunt and co-workers also quantified mast cells in the peribronchiolar tissue of IPF and normal human lung by using rabbit antiserum to human mast cell tryptase. They reported over a twofold increase in the connective tissue directly adjacent to the lumen of small airways and other fibrotic foci in IPF versus control lungs, which appeared to be actively degranulating (Hunt et al., 1992). In addition, Edwards et al. (2005) profiled c-kit expressing cells in interstitial lung disease (including IPF), reporting elevated c-kit+ mast cells both positive for the serine proteases chymase and tryptase, as well as MMP expression in disease versus normal lung environment. Pesci et al. (1993) did not include IPF in their patient population, they did evaluate the numbers of mast cells in the fibrotic lung disorders in order to determine their role in the pathogenesis of this disorder and reported a clear increase in mast cell number which significantly correlated with the degree of fibrosis. The presence of the mast cell mediator basic FGF has also been evaluated in human interstitial lung disease to determine if mast cells and their contents drive the fibrotic response (Inoue et al., 1993). Analysis of lung tissue, BAL fluid, and serum from patients with IPF detected increased bFGF+ MC in the lung interstitium, correlating with the distribution of ECM deposition and the extent of fibrosis morphometrically, as well as elevated bFGF in the BAL fluid (Inoue et al., 1993). Moreover, increased levels of tryptase were measured in the BAL fluid of IPF patients, with tryptase-positive IPF cases reported to have a poorer outcome (Kawatani et al., 2007).

More recently Andersson and colleagues investigated the distribution pattern and phenotypes of lung mast cells in fibrosis-related conditions. As detailed earlier, mast cell subtypes can be broadly identified by their granule content, being either typtase and chymase positive (MC_TC_ or CTMCs), or tryptase positive but chymase negative (MC_T_ orMMCs). MMCs are most frequently found in the healthy lung (Metcalfe et al., 1997). They reported that a significantly elevated number of TGFβ+ mast cells in fibrotic areas of the alveolar parenchyma in patients with IPF, however no change was detected in the small airways and were decreased in the pulmonary vessels. The authors also reported an increase in mast cell expression of TGF-β in the increased mast cells found in the alveolar parenchyma, and that the density and percentage of MCTC correlated positively with the degree of fibrosis and negatively with patient lung function (Andersson et al., 2011b). Increased activated mast cells were also reported in close proximity to fibroblast foci and alveolar type II cells in the IPF lung, as well as elevated mast cell tryptase in patients with IPF (Wygrecka et al., 2013). However, Cha et al. (2012) recently reported that although chymase positive mast cells are elevated in IPF, this correlated with a slower rate of decline in forced vital capacity suggesting a protective mechanism.

A number of experimental animal studies have now been reported to investigate the role mast cells play in the lung, typically using mast cell-deficient strains genetically engineered through a genetic deficient in c-kit. Early studies in WBB6F1-W/Wv mice reported bleomycin-induced fibrosis regardless of the mast cell deficiency (Mori et al., 1991). Additionally bleomycin could induce fibrosis using Ws/Ws mast cell-deficient rats to a similar extent as that observed in wild types (Okazaki et al., 1998). However, more recently Veerappan et al. (2013) reported a pivotal role of mast cells in the initiating lung fibrosis. In this study, the authors administered bleomycin to mast-cell-deficient WBB6F1-W/Wv mice and their respective controls and demonstrated a protection, which was lost post restoration of the mast cell population (Veerappan et al., 2013). The authors postulate that the protective effects seen in their studies compared to the earlier studies (Mori et al., 1991; Okazaki et al., 1998), may be due to differences in experimental protocol (theirs being a 2 week model vs a 5 week model used by others). In a recent pharmacological study, berberine, a plant alkaloid was given to mice exposed to bleomycin. In this study, bleomycin induced mast cell accumulation in the lung and increased histamine levels. Berberine significantly blocked collagen accumulation as observed by a reduction in the hydroxyproline level, which corresponded with reduced histamine levels (Chitra et al., 2013), supporting a role for mast cell in the pathogenesis of lung disease.

The underlying mechanism for increased mast cells in IPF remains unknown, however a role for SCF is emerging. SCF is expressed as soluble or membrane-bound forms and promotes survival, proliferation, mobilization from the bone marrow and adhesion of hematopoietic stem cells and other progenitor cells through binding with its receptor, c-kit (Fleming et al., 1993; Lukacs et al., 1996; Domen and Weissman, 2000; Nakamura et al., 2004). In the lung this axis has been shown to be important in fibrosis as suggested by increased secretion of SCF from alveolar fibroblasts from patients with diffuse interstitial fibrosis (Fireman et al., 1999). In a mouse model of cockroach induced asthma, blockade of SCF has been shown to attenuate airway remodeling and collagen deposition in the lung (Dolgachev et al., 2009). In IPF, Wygrecka and colleagues demonstrated increased expression of membrane SCF in the IPF lung and in fibroblasts isolated from IPF tissue. Co-culture of lung fibroblasts from IPF patients with mast cells enhanced MC survival and proliferation, an effect dependent on SCF and c-kit (Wygrecka et al., 2013). This phenomenon has also been reported elsewhere, whereby SCF expressed on bronchial airway smooth muscle induces the survival, proliferation and activation of lung mast cells (Hollins et al., 2008). Additionally, blockade of SCF either genetically or using an antibody approach abrogates bleomycin-induced lung fibrosis, although mast cell measurements were not made in this model (Ding et al., 2013).

## SUMMARY AND FUTURE CLINICAL DIRECTIONS

Idiopathic pulmonary fibrosis is a devastating disease for the patient. There is currently no effective treatment for this disease and the prognosis is bleak. As the term “idiopathic” indicates, the causes of the disease are unknown, as are the molecular mechanisms underpinning initiation and progression of the condition. Clearly, fibrotic processes play a key role in driving the relentless destruction of alveolar integrity, resulting eventually in a declining ability of the lung to oxygenate the blood. This decline is the root cause of the deteriorating health of the patient once the disease passes from its typically undiagnosed, early phase, into its clinically symptomatic phase. Within less than 3 years in most patients have lost much of their respiratory capacity and require drastic measures to survive.

The pharmaceutical and biotechnology industry has made many attempts to find effective treatments for IPF, but the disease has so far defied all attempts at therapeutic intervention. Clinical trial failures may arise for many reasons, including disease heterogeneity, lack of readily measurable clinical end points other than overall survival, and, perhaps most of all, a lack of understanding of the underlying molecular mechanisms of the progression of IPF.

On the positive side, with emerging new insights into the pathways and cell types involved in IPF come new opportunities for therapeutic intervention. Technologies for molecular profiling of patient tissue samples are already revealing many hitherto unexpected aspects of the disease pathology. Several new cell types, including the myofibroblast and the mast cell, offer therapeutic possibilities not previously exploited. However, the only conclusive way to determine if these cells are important for the pathogenesis of IPF is to target them with sufficiently powerful therapeutics and determine the impact on disease progression in phase 2 clinical trials. Another potentially helpful way to success may be that new therapeutics are first tested in other fibrotic conditions than IPF. Based on our current understanding of disease mechanisms, it appears likely that therapeutic interventions that are efficacious in one form of fibrotic disease will be efficacious in other fibrotic conditions. Thus, the clinically most feasible disease indication may serve as a first read-out to support the testing in more challenging indications, such as IPF. This may well be the case for mast cell-targeted therapies. We postulate that this hypothesis should be tested.

## Conflict of Interest Statement

The authors declare that the research was conducted in the absence of any commercial or financial relationships that could be construed as a potential conflict of interest. The authors and editor declare that while they are currently employed by the same institution there was no conflict of interest during the review and handling of this manuscript.
